# Supplementary material for: Multi-Omics Analysis of Transcriptomic and Metabolomics Profiles Reveal the Molecular Regulatory Network of Marbling in Early Castrated Holstein Steers
Source: Animals (Basel). 2022 Dec 2;12(23):3398. doi: 10.3390/ani12233398 (PMC9736081; doi:10.3390/ani12233398)
Supplement: Supplementary file 1 [file animals-12-03398-s001.zip › animals-2004272-supplementary.pdf]

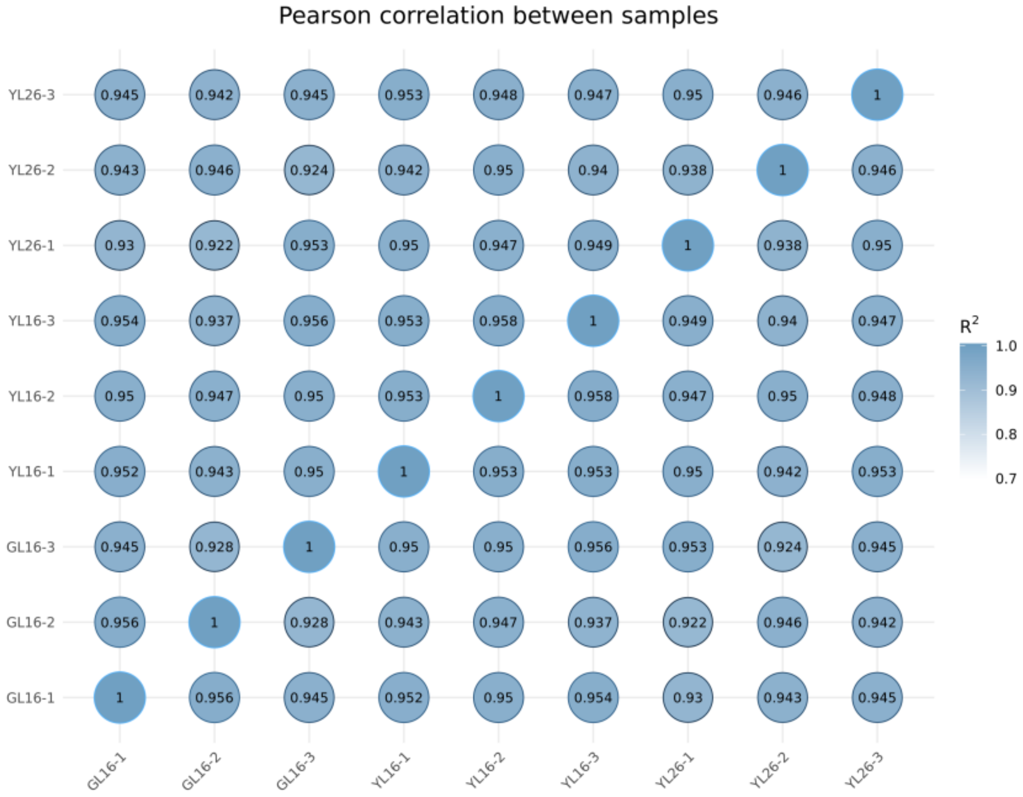

Figure S1. Heat map of correlation coefficient between samples.

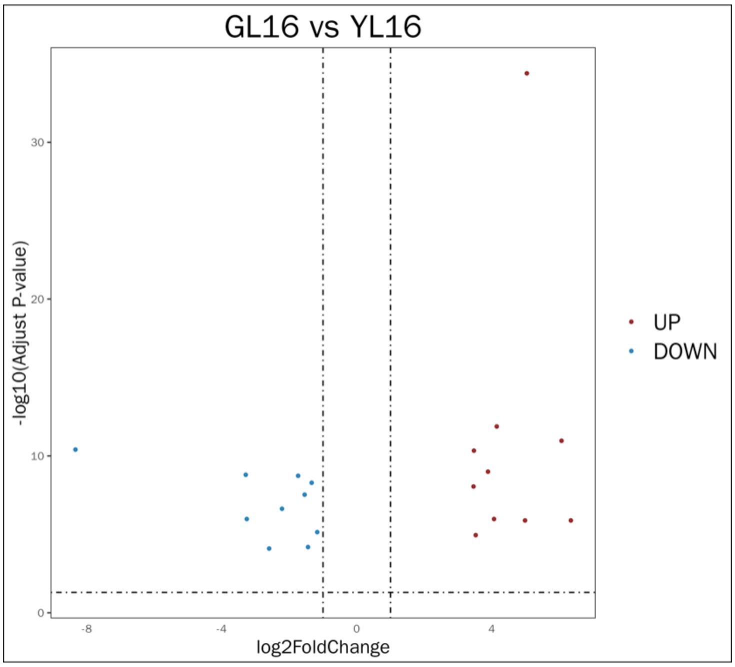

(A)

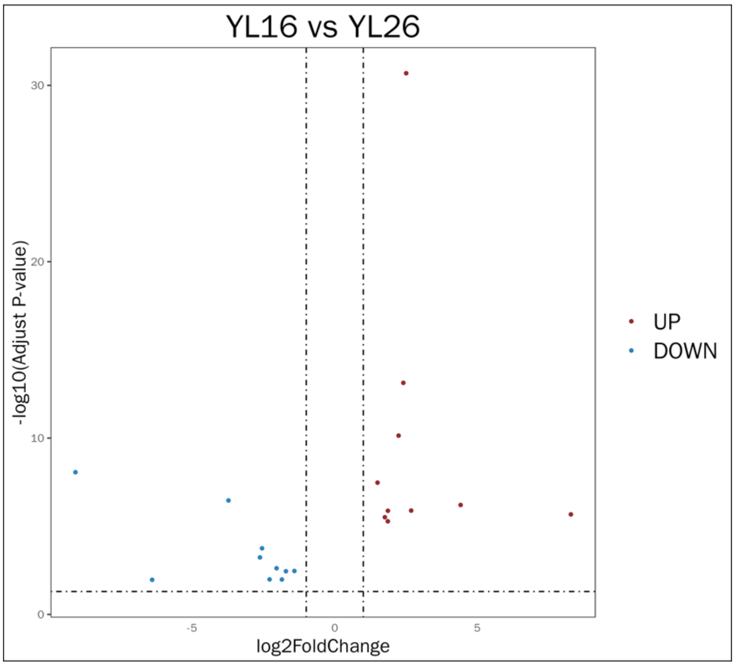

(B)

Figure S2. Different expressed genes between GL16 and YL16 (A) or YL16 and YL26 (B). The red points indicate significantly upregulated expressed genes and the green points indicated significantly downregulated expressed genes.

GL16 = non-castrated and slaughtered at 16 months of age; YL16 = castrated at birth and slaughtered at 16 months of age; YL26 = castrated at birth and slaughtered at 26 months of age.

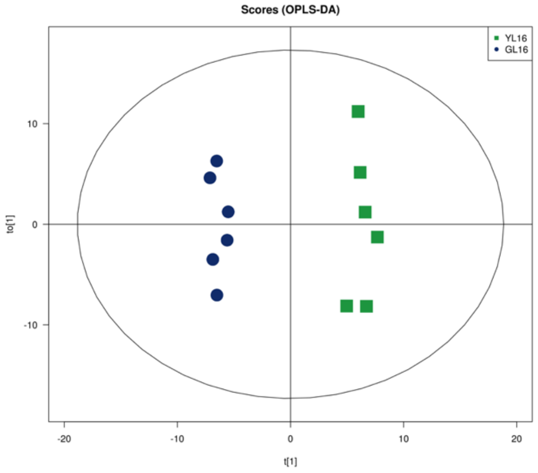

(A)

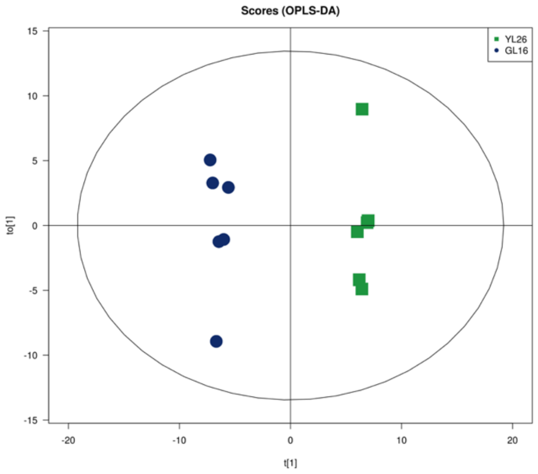

(B)

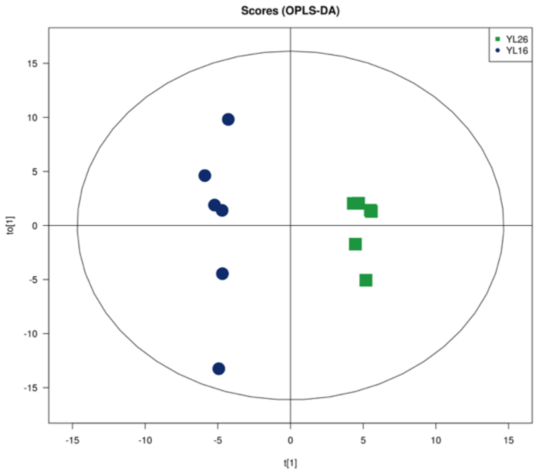

Scores (OPLS-DA)
YL26
YL16
to[1]
t[1]

(C)

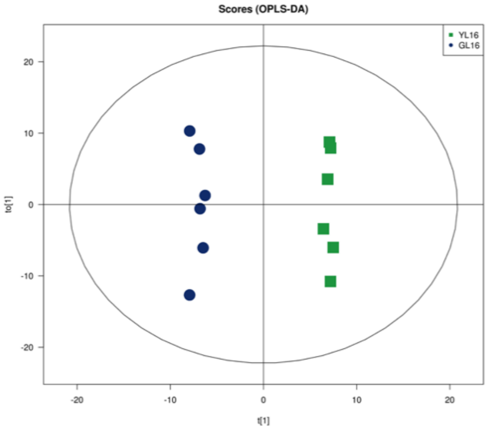

Scores (OPLS-DA)
YL16
GL16
to[1]
t[1]

(D)

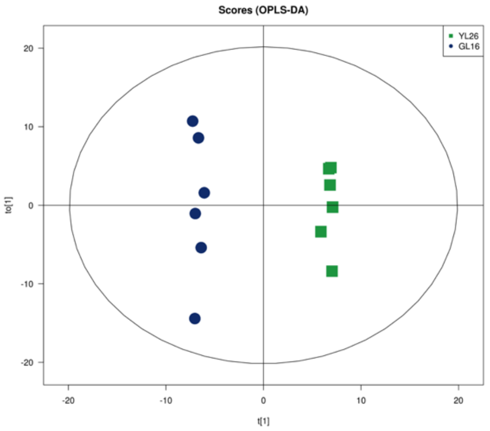

Scores (OPLS-DA)
YL26
GL16
to[1]
t[1]

(E)

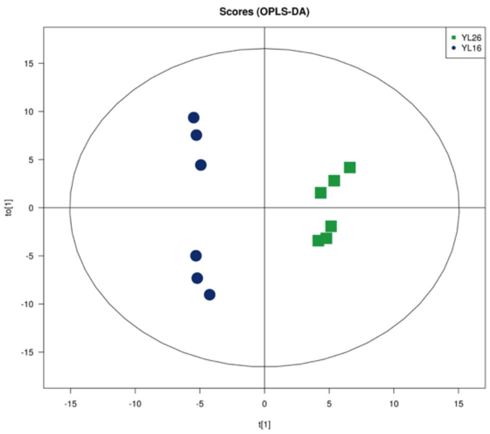

(F)

Figure S3. Score plot of orthogonal partial-least-squares discriminant analysis (OPLS- DA) model obtained for YL16 and GL16 (A), YL26 and GL16 (B), and YL26 and YL16 (C) in positive mode; and for YL16 and GL16 (D), YL26 and GL16 (E), and YL26 and YL16 (F) in negative mode. GL16 = non-castrated and slaughtered at 16 months of age; YL16 = castrated at birth and slaughtered at 16 months of age; YL26 = castrated at birth and slaughtered at 26 months of age.

Table S1. List of data quality of each sample.

**Sample** [1]	**Raw Reads**	**Clean Reads**	**Clean Bases**	**Q 20 (%)**	**Q 30 (%)**	**GC (%)**
GL16-1	47596226	47247592	6.57G	98.36	94.7	46.18
GL16-2	44969762	44553856	6.2G	98.14	94.24	46.97
GL16-3	45161024	44634590	6.19G	98.32	94.68	44.04
YL16-1	45251850	44764350	6.23G	98.17	94.31	44.95
YL16-2	43835412	43356944	6.03G	98.24	94.44	45.95
YL16-3	40522788	40147578	5.59G	98.14	94.2	45.18
YL26-1	41569262	41225262	5.75G	98.23	94.41	43.81
YL26-2	43561908	43228308	6.01G	98.18	94.28	46.64
YL26-3	47066668	46676984	6.5G	98.41	94.88	45.84

[1] GL16 = non-castrated and slaughtered at 16 months of age; YL16 = castrated at birth and slaughtered at 16 months of age; YL26 = castrated at birth and slaughtered at 26 months of age.

Table S2. Table of comparison between reads and reference genome.

Sample [1]	Total Reads	Total Mapped	Multiple Mapped	Unique Mapped	Reads Mapped to '+'	Reads Mapped to '-'	Non-Splice Reads	Splice Reads
GL16-1	47247592	46135740 (97.65%)	1252659 (2.65%)	44883081 (95.00%)	22428045 (47.47%)	22455036 (47.53%)	30778640 (65.14%)	14104441 (29.85%)
GL16-2	44553856	43427641 (97.47)	1471504 (3.30%)	41956137 (94.17%)	20966268 (47.06%)	20989869 (47.11%)	25178063 (56.51%)	16778074 (37.66%)
GL16-3	44634590	43259961 (96.92%)	925251 (2.07%)	42334710 (94.85%)	21160717 (47.41%)	21173993 (47.44%)	32631000 (73.11%)	9703710 (21.74%)
YL16-1	44764350	43485230 (97.14%)	1058469 (2.36%)	42426761 (94.78%)	21199350 (47.36)	21227411 (47.42%)	30854307 (68.93%)	11572454 (25.85%)
YL16-2	43356944	42264189 (97.48%)	1078730 (2.49%)	41185459 (94.99%)	20585168 (47.48%)	20600291 (47.51%)	27400226 (63.20%)	13785233 (31.79%)
YL16-3	40147578	39097891 (97.39%)	949828 (2.37%)	38148063 (95.02%)	19061973 (47.48%)	19086090 (47.54%)	26938932 (67.10%)	11209131 (27.92%)
YL26-1	41225262	40074116 (97.21%)	853349 (2.07%)	39220767 (95.14%)	19597256 (47.54%)	19623511 (47.60%)	28941145 (70.20%)	10279622 (24.94%)
YL26-2	43228308	42071191 (97.32%)	1265163 (2.93%)	40806028 (94.40%)	20396106 (47.18%)	20409922 (47.21%)	24683947 (57.10%)	16122081 (37.30%)
YL26-3	46676984	45124242 (96.67%)	1200836 (2.57%)	43923406 (94.10%)	21952728 (47.03%)	21970678 (47.07%)	30449228 (65.23%)	13474178 (28.87%)

[1] GL16 = non-castrated and slaughtered at 16 months of age; YL16 = castrated at birth and slaughtered at 16 months of age; YL26 = castrated at birth and slaughtered at 26 months of age.

Table S3-1. GL16 vs. YL16 upregulated pathways (KEGG).

ID	Description	Test	Test All	Ref	Ref All	*p*-Value	FDR	Rich Factor	Gene_id	KEGG_id	URL
bta04010	MAPK signaling pathway	2	6	287	8062	0.017229	0.085823	0.006969	ENSBTAG00000004587|ENSBTAG00000030259	bta:515310|bta:524957	http://www.kegg.jp/kegg-bin/show_pathway?bta04010+bta:515310+bta:524957
bta04614	Renin–angiotensin system	1	6	25	8062	0.018468	0.085823	0.04	ENSBTAG00000034402	bta:509235	http://www.kegg.jp/kegg-bin/show_pathway?bta04614+bta:509235
bta00040	Pentose and glucuronate interconversions	1	6	29	8062	0.021396	0.085823	0.034483	ENSBTAG00000001582	bta:525193	http://www.kegg.jp/kegg-bin/show_pathway?bta00040+bta:525193
bta00052	Galactose metabolism	1	6	29	8062	0.021396	0.085823	0.034483	ENSBTAG00000001582	bta:525193	http://www.kegg.jp/kegg-bin/show_pathway?bta00052+bta:525193
bta00591	Linoleic acid metabolism	1	6	32	8062	0.023588	0.085823	0.03125	ENSBTAG00000049496	bta:785540	http://www.kegg.jp/kegg-bin/show_pathway?bta00591+bta:785540
bta00051	Fructose and mannose metabolism	1	6	33	8062	0.024317	0.085823	0.030303	ENSBTAG00000001582	bta:525193	http://www.kegg.jp/kegg-bin/show_pathway?bta00051+bta:525193
bta00790	Folate biosynthesis	1	6	37	8062	0.027231	0.085823	0.027027	ENSBTAG00000001582	bta:525193	http://www.kegg.jp/kegg-bin/show_pathway?bta00790+bta:525193
bta04340	Hedgehog signaling pathway	1	6	50	8062	0.036651	0.085823	0.02	ENSBTAG00000006631	bta:517588	http://www.kegg.jp/kegg-bin/show_pathway?bta04340+bta:517588
bta00561	Glycerolipid metabolism	1	6	60	8062	0.043845	0.085823	0.016667	ENSBTAG00000001582	bta:525193	http://www.kegg.jp/kegg-bin/show_pathway?bta00561+bta:525193
bta05217	Basal cell carcinoma	1	6	62	8062	0.045278	0.085823	0.016129	ENSBTAG00000006631	bta:517588	http://www.kegg.jp/kegg-bin/show_pathway?bta05217+bta:517588
bta00830	Retinol metabolism	1	6	63	8062	0.045994	0.085823	0.015873	ENSBTAG00000049496	bta:785540	http://www.kegg.jp/kegg-bin/show_pathwa

											y?bta00830+bta:785540
bta05221	Acute myeloid leukemia	1	6	66	8062	0.04814	0.085823	0.015152	ENSBTAG00000004587	bta:515310	http://www.kegg.jp/kegg-bin/show_pathway?bta05221+bta:515310
bta00140	Steroid hormone biosynthesis	1	6	68	8062	0.049568	0.085823	0.014706	ENSBTAG00000049496	bta:785540	http://www.kegg.jp/kegg-bin/show_pathway?bta00140+bta:785540
bta05204	Chemical carcinogenesis	1	6	74	8062	0.053841	0.085823	0.013514	ENSBTAG00000049496	bta:785540	http://www.kegg.jp/kegg-bin/show_pathway?bta05204+bta:785540
bta00590	Arachidonic acid metabolism	1	6	77	8062	0.055972	0.085823	0.012987	ENSBTAG00000049496	bta:785540	http://www.kegg.jp/kegg-bin/show_pathway?bta00590+bta:785540
bta04750	Inflammatory mediator regulation of TRP channels	1	6	97	8062	0.070075	0.100732	0.010309	ENSBTAG00000049496	bta:785540	http://www.kegg.jp/kegg-bin/show_pathway?bta04750+bta:785540
bta04974	Protein digestion and absorption	1	6	108	8062	0.077756	0.102907	0.009259	ENSBTAG00000034402	bta:509235	http://www.kegg.jp/kegg-bin/show_pathway?bta04974+bta:509235
bta04726	Serotonergic synapse	1	6	112	8062	0.080536	0.102907	0.008929	ENSBTAG00000049496	bta:785540	http://www.kegg.jp/kegg-bin/show_pathway?bta04726+bta:785540
bta05202	Transcriptional misregulation in cancer	1	6	191	8062	0.13403	0.162246	0.005236	ENSBTAG00000004587	bta:515310	http://www.kegg.jp/kegg-bin/show_pathway?bta05202+bta:515310
bta04024	cAMP signaling pathway	1	6	218	8062	0.151706	0.174462	0.004587	ENSBTAG00000006631	bta:517588	http://www.kegg.jp/kegg-bin/show_pathway?bta04024+bta:517588
bta04014	Ras signaling pathway	1	6	230	8062	0.159465	0.174653	0.004348	ENSBTAG00000030259	bta:524957	http://www.kegg.jp/kegg-bin/show_pathway?bta04014+bta:524957
bta01100	Metabolic pathways	2	6	1480	8062	0.304325	0.318158	0.001351	ENSBTAG00000001582|ENSBTAG00000049496	bta:525193|bta:785540	http://www.kegg.jp/kegg-bin/show_pathway?bta01100+bta:525193+bta:785540

bta05200	Pathways in cancer	1	6	517	8062	0.328195	0.328195	0.001934	ENSBTAG00000006631	bta:517588	http://www.kegg.jp/kegg-bin/show_pathway?bta05200+bta:517588

GL16 = non-castrated and slaughtered at 16 months of age; YL16 = castrated at birth and slaughtered at 16 months of age.

Table S3-2. GL16 vs. YL16 downregulated pathways (KEGG).

ID	Description	Test	Test All	Ref	Ref All	*p*-Value	FDR	Rich Factor	Gene_id	KEGG_id	URL
bta04064	NF-kappa B signaling pathway	4	15	105	8062	3.32E-05	0.003055	0.038095	ENSBTAG00000003033丨ENSBTAG00000016683丨ENSBTAG00000025462丨ENSBTAG00000037778	bta:504939丨bta:282291丨bta:618405丨bta:613667	http://www.kegg.jp/kegg-bin/show_pathway?bta04064+bta:504939+bta:282291+bta:618405+bta:613667
bta05220	Chronic myeloid leukemia	3	15	76	8062	0.000338	0.015533	0.039474	ENSBTAG00000003033丨ENSBTAG00000016683丨ENSBTAG00000025462	bta:504939丨bta:282291丨bta:618405	http://www.kegg.jp/kegg-bin/show_pathway?bta05220+bta:504939+bta:282291+bta:618405
bta05222	Small cell lung cancer	3	15	91	8062	0.000574	0.017597	0.032967	ENSBTAG00000003033丨ENSBTAG00000016683丨ENSBTAG00000025462	bta:504939丨bta:282291丨bta:618405	http://www.kegg.jp/kegg-bin/show_pathway?bta05222+bta:504939+bta:282291+bta:618405
bta00591	Linoleic acid metabolism	2	15	32	8062	0.001552	0.030684	0.0625	ENSBTAG00000018365丨ENSBTAG00000052665	bta:282213丨bta:507988	http://www.kegg.jp/kegg-bin/show_pathway?bta00591+bta:282213+bta:507988
bta05216	Thyroid cancer	2	15	36	8062	0.001963	0.030684	0.055556	ENSBTAG00000003033丨ENSBTAG00000025462	bta:504939丨bta:618405	http://www.kegg.jp/kegg-bin/show_pathway?bta05216+bta:504939+bta:618405

bta04210	Apoptosis	3	15	140	8062	0.002001	0.030684	0.021429	ENSBTAG00000003033|ENSBTAG00000016683|ENSBTAG00000025462	bta:504939|bta:282291|bta:618405	http://www.kegg.jp/kegg-bin/show_pathway?bta04210+bta:504939+bta:282291+bta:618405
bta05134	Legionellosis	2	15	56	8062	0.004696	0.047672	0.035714	ENSBTAG00000016683|ENSBTAG00000037778	bta:282291|bta:613667	http://www.kegg.jp/kegg-bin/show_pathway?bta05134+bta:282291+bta:613667
bta05213	Endometrial cancer	2	15	57	8062	0.004861	0.047672	0.035088	ENSBTAG00000003033|ENSBTAG00000025462	bta:504939|bta:618405	http://www.kegg.jp/kegg-bin/show_pathway?bta05213+bta:504939+bta:618405
bta05217	Basal cell carcinoma	2	15	62	8062	0.005729	0.047672	0.032258	ENSBTAG00000003033|ENSBTAG00000025462	bta:504939|bta:618405	http://www.kegg.jp/kegg-bin/show_pathway?bta05217+bta:504939+bta:618405
bta05169	Epstein–Barr virus infection	3	15	212	8062	0.006457	0.047672	0.014151	ENSBTAG00000003033|ENSBTAG00000016683|ENSBTAG00000025462	bta:504939|bta:282291|bta:618405	http://www.kegg.jp/kegg-bin/show_pathway?bta05169+bta:504939+bta:282291+bta:618405
bta05223	Non-small-cell lung cancer	2	15	67	8062	0.006663	0.047672	0.029851	ENSBTAG00000003033|ENSBTAG00000025462	bta:504939|bta:618405	http://www.kegg.jp/kegg-bin/show_pathway?bta05223+bta:504939+bta:618405
bta00140	Steroid hormone biosynthesis	2	15	68	8062	0.006857	0.047672	0.029412	ENSBTAG00000018365|ENSBTAG00000052665	bta:282213|bta:507988	http://www.kegg.jp/kegg-bin/show_pathway?bta00140+bta:282213+bta:507988

bta05218	Melanoma	2	15	70	8062	0.007254	0.047672	0.028571	ENSBTAG00000003033丨ENSBTAG00000025462	bta:504939丨bta:618405	http://www.kegg.jp/kegg-bin/show_pathway?bta05218+bta:504939+bta:618405
bta05214	Glioma	2	15	73	8062	0.007868	0.047672	0.027397	ENSBTAG00000003033丨ENSBTAG00000025462	bta:504939丨bta:618405	http://www.kegg.jp/kegg-bin/show_pathway?bta05214+bta:504939+bta:618405
bta05204	Chemical carcinogenesis	2	15	74	8062	0.008078	0.047672	0.027027	ENSBTAG00000018365丨ENSBTAG00000052665	bta:282213丨bta:507988	http://www.kegg.jp/kegg-bin/show_pathway?bta05204+bta:282213+bta:507988
bta05212	Pancreatic cancer	2	15	75	8062	0.008291	0.047672	0.026667	ENSBTAG00000003033丨ENSBTAG00000025462	bta:504939丨bta:618405	http://www.kegg.jp/kegg-bin/show_pathway?bta05212+bta:504939+bta:618405
bta04115	p53 signaling pathway	2	15	79	8062	0.009166	0.049602	0.025316	ENSBTAG00000003033丨ENSBTAG00000025462	bta:504939丨bta:618405	http://www.kegg.jp/kegg-bin/show_pathway?bta04115+bta:504939+bta:618405
bta05210	Colorectal cancer	2	15	87	8062	0.011034	0.056396	0.022989	ENSBTAG00000003033丨ENSBTAG00000025462	bta:504939丨bta:618405	http://www.kegg.jp/kegg-bin/show_pathway?bta05210+bta:504939+bta:618405
bta04657	IL-17 signaling pathway	2	15	90	8062	0.011775	0.057015	0.022222	ENSBTAG00000016683丨ENSBTAG00000037778	bta:282291丨bta:613667	http://www.kegg.jp/kegg-bin/show_pathway?bta04657+bta:282291+bta:613667
bta04668	TNF signaling pathway	2	15	119	8062	0.020011	0.090456	0.016807	ENSBTAG00000016683丨ENSBTAG00000037778	bta:282291丨bta:613667	http://www.kegg.jp/kegg-bin/show_pathway?bta04668+bta:282291+bta:613667

bta04110	Cell cycle	2	15	121	8062	0.020648	0.090456	0.016529	ENSBTAG00000003033|ENSBTAG00000025462	bta:504939|bta:618405	http://www.kegg.jp/kegg-bin/show_pathway?bta04110+bta:504939+bta:618405
bta04068	FoxO signaling pathway	2	15	127	8062	0.022609	0.094548	0.015748	ENSBTAG00000003033|ENSBTAG00000025462	bta:504939|bta:618405	http://www.kegg.jp/kegg-bin/show_pathway?bta04068+bta:504939+bta:618405
bta05224	Breast cancer	2	15	146	8062	0.02931	0.114786	0.013699	ENSBTAG00000003033|ENSBTAG00000025462	bta:504939|bta:618405	http://www.kegg.jp/kegg-bin/show_pathway?bta05224+bta:504939+bta:618405
bta05226	Gastric cancer	2	15	149	8062	0.030433	0.114786	0.013423	ENSBTAG00000003033|ENSBTAG00000025462	bta:504939|bta:618405	http://www.kegg.jp/kegg-bin/show_pathway?bta05226+bta:504939+bta:618405
bta04218	Cellular senescence	2	15	151	8062	0.031192	0.114786	0.013245	ENSBTAG00000003033|ENSBTAG00000025462	bta:504939|bta:618405	http://www.kegg.jp/kegg-bin/show_pathway?bta04218+bta:504939+bta:618405
bta05225	Hepatocellular carcinoma	2	15	170	8062	0.03877	0.136386	0.011765	ENSBTAG00000003033|ENSBTAG00000025462	bta:504939|bta:618405	http://www.kegg.jp/kegg-bin/show_pathway?bta05225+bta:504939+bta:618405
bta04621	NOD-like receptor signaling pathway	2	15	173	8062	0.040026	0.136386	0.011561	ENSBTAG00000016683|ENSBTAG00000037778	bta:282291|bta:613667	http://www.kegg.jp/kegg-bin/show_pathway?bta04621+bta:282291+bta:613667
bta04062	Chemokine signaling pathway	2	15	183	8062	0.044326	0.144526	0.010929	ENSBTAG00000016683|ENSBTAG00000037778	bta:282291|bta:613667	http://www.kegg.jp/kegg-bin/show_pathway?bta04062+bta:282291+bta:613667

bta00592	Alpha-linolenic acid metabolism	1	15	25	8062	0.045557	0.144526	0.04	ENSBTAG00000015505	bta:521822	http://www.kegg.jp/kegg-bin/show_pathway?bta00592+bta:521822
bta05202	Transcriptional misregulation in cancer	2	15	191	8062	0.047887	0.144808	0.010471	ENSBTAG00000003033丨ENSBTAG00000025462	bta:504939丨bta:618405	http://www.kegg.jp/kegg-bin/show_pathway?bta05202+bta:504939+bta:618405
bta05167	Kaposi's sarcoma-associated herpesvirus infection	2	15	193	8062	0.048794	0.144808	0.010363	ENSBTAG00000016683丨ENSBTAG00000037778	bta:282291丨bta:613667	http://www.kegg.jp/kegg-bin/show_pathway?bta05167+bta:282291+bta:613667
bta01040	Biosynthesis of unsaturated fatty acids	1	15	29	8062	0.052664	0.151408	0.034483	ENSBTAG00000015505	bta:521822	http://www.kegg.jp/kegg-bin/show_pathway?bta01040+bta:521822
bta05132	Salmonella infection	2	15	213	8062	0.058206	0.162272	0.00939	ENSBTAG00000013468丨ENSBTAG00000016683	bta:531336丨bta:282291	http://www.kegg.jp/kegg-bin/show_pathway?bta05132+bta:531336+bta:282291
bta05200	Pathways in cancer	3	15	517	8062	0.06702	0.181347	0.005803	ENSBTAG00000003033丨ENSBTAG00000016683丨ENSBTAG00000025462	bta:504939丨bta:282291丨bta:618405	http://www.kegg.jp/kegg-bin/show_pathway?bta05200+bta:504939+bta:282291+bta:618405
bta04973	Carbohydrate digestion and absorption	1	15	42	8062	0.075421	0.19825	0.02381	ENSBTAG00000012851	bta:282361	http://www.kegg.jp/kegg-bin/show_pathway?bta04973+bta:282361
bta04930	Type II diabetes mellitus	1	15	44	8062	0.078877	0.201574	0.022727	ENSBTAG00000012007	bta:338437	http://www.kegg.jp/kegg-bin/show_pathway?bta04930+bta:338437

bta00565	Ether lipid metabolism	1	15	48	8062	0.085752	0.21322	0.020833	ENSBTAG00000019315	bta:282311	http://www.kegg.jp/kegg-bin/show_pathway?bta00565+bta:282311
bta04978	Mineral absorption	1	15	55	8062	0.097667	0.225747	0.018182	ENSBTAG00000012851	bta:282361	http://www.kegg.jp/kegg-bin/show_pathway?bta04978+bta:282361
bta04010	MAPK signaling pathway	2	15	287	8062	0.097805	0.225747	0.006969	ENSBTAG00000003033|ENSBTAG00000025462	bta:504939|bta:618405	http://www.kegg.jp/kegg-bin/show_pathway?bta04010+bta:504939+bta:618405
bta01212	Fatty acid metabolism	1	15	56	8062	0.099358	0.225747	0.017857	ENSBTAG00000015505	bta:521822	http://www.kegg.jp/kegg-bin/show_pathway?bta01212+bta:521822
bta00982	Drug metabolism — cytochrome P450	1	15	57	8062	0.101045	0.225747	0.017544	ENSBTAG00000018365	bta:282213	http://www.kegg.jp/kegg-bin/show_pathway?bta00982+bta:282213
bta05168	Herpes simplex virus 1 infection	2	15	296	8062	0.103058	0.225747	0.006757	ENSBTAG00000016683|ENSBTAG00000000943	bta:282291|bta:789857	http://www.kegg.jp/kegg-bin/show_pathway?bta05168+bta:282291+bta:789857
bta04623	Cytosolic DNA-sensing pathway	1	15	61	8062	0.107766	0.227154	0.016393	ENSBTAG00000016683	bta:282291	http://www.kegg.jp/kegg-bin/show_pathway?bta04623+bta:282291
bta00830	Retinol metabolism	1	15	63	8062	0.111108	0.227154	0.015873	ENSBTAG00000052665	bta:507988	http://www.kegg.jp/kegg-bin/show_pathway?bta00830+bta:507988
bta00980	Metabolism of xenobiotics by	1	15	63	8062	0.111108	0.227154	0.015873	ENSBTAG00000018365	bta:282213	http://www.kegg.jp/kegg-bin/show_pathway?bta00980+bta:282213

	cytochrome P450									
bta04920	Adipocytokine signaling pathway	1	15	68	8062	0.119413	0.238827	0.014706	ENSBTAG00000016683	bta:282291	http://www.kegg.jp/kegg-bin/show_pathway?bta04920+bta:282291
bta00983	Drug metabolism—other enzymes	1	15	72	8062	0.126005	0.24151	0.013889	ENSBTAG00000018365	bta:282213	http://www.kegg.jp/kegg-bin/show_pathway?bta00983+bta:282213
bta04662	B-cell receptor signaling pathway	1	15	72	8062	0.126005	0.24151	0.013889	ENSBTAG00000016683	bta:282291	http://www.kegg.jp/kegg-bin/show_pathway?bta04662+bta:282291
bta05140	Leishmaniasis	1	15	75	8062	0.130919	0.243147	0.013333	ENSBTAG00000016683	bta:282291	http://www.kegg.jp/kegg-bin/show_pathway?bta05140+bta:282291
bta00590	Arachidonic acid metabolism	1	15	77	8062	0.134181	0.243147	0.012987	ENSBTAG00000018365	bta:282213	http://www.kegg.jp/kegg-bin/show_pathway?bta00590+bta:282213
bta03320	PPAR signaling pathway	1	15	79	8062	0.137431	0.243147	0.012658	ENSBTAG00000015505	bta:521822	http://www.kegg.jp/kegg-bin/show_pathway?bta03320+bta:521822
bta04622	RIG-I-like receptor signaling pathway	1	15	79	8062	0.137431	0.243147	0.012658	ENSBTAG00000016683	bta:282291	http://www.kegg.jp/kegg-bin/show_pathway?bta04622+bta:282291
bta04917	Prolactin signaling pathway	1	15	83	8062	0.143897	0.249783	0.012048	ENSBTAG00000012007	bta:338437	http://www.kegg.jp/kegg-bin/show_pathway?bta04917+bta:338437
bta05235	PD-L1 expression and PD-1 checkpoint	1	15	89	8062	0.153511	0.25837	0.011236	ENSBTAG00000016683	bta:282291	http://www.kegg.jp/kegg-bin/show_pathway?bta05235+bta:282291

	pathway in cancer										
bta04061	Viral protein interaction with cytokine and cytokine receptor	1	15	91	8062	0.156693	0.25837	0.010989	ENSBTAG00000037778	bta:613667	http://www.kegg.jp/kegg-bin/show_pathway?bta04061+bta:613667
bta04976	Bile secretion	1	15	93	8062	0.159864	0.25837	0.010753	ENSBTAG00000012851	bta:282361	http://www.kegg.jp/kegg-bin/show_pathway?bta04976+bta:282361
bta04658	Th1 and Th2 cell differentiation	1	15	95	8062	0.163024	0.25837	0.010526	ENSBTAG00000016683	bta:282291	http://www.kegg.jp/kegg-bin/show_pathway?bta04658+bta:282291
bta05215	Prostate cancer	1	15	96	8062	0.1646	0.25837	0.010417	ENSBTAG00000016683	bta:282291	http://www.kegg.jp/kegg-bin/show_pathway?bta05215+bta:282291
bta05323	Rheumatoid arthritis	1	15	97	8062	0.166173	0.25837	0.010309	ENSBTAG00000037778	bta:613667	http://www.kegg.jp/kegg-bin/show_pathway?bta05323+bta:613667
bta04625	C-type lectin receptor signaling pathway	1	15	100	8062	0.170876	0.25837	0.01	ENSBTAG00000016683	bta:282291	http://www.kegg.jp/kegg-bin/show_pathway?bta04625+bta:282291
bta04620	Toll-like receptor signaling pathway	1	15	103	8062	0.175554	0.25837	0.009709	ENSBTAG00000016683	bta:282291	http://www.kegg.jp/kegg-bin/show_pathway?bta04620+bta:282291
bta04660	T-cell receptor signaling pathway	1	15	105	8062	0.178659	0.25837	0.009524	ENSBTAG00000016683	bta:282291	http://www.kegg.jp/kegg-bin/show_pathway?bta04660+bta:282291

bta05146	Amoebiasis	1	15	106	8062	0.180207	0.25837	0.009434	ENSBTAG00000037778	bta:613667	http://www.kegg.jp/kegg-bin/show_pathway?bta05146+bta:613667
bta04931	Insulin resistance	1	15	107	8062	0.181753	0.25837	0.009346	ENSBTAG00000016683	bta:282291	http://www.kegg.jp/kegg-bin/show_pathway?bta04931+bta:282291
bta04659	Th17 cell differentiation	1	15	110	8062	0.186373	0.25837	0.009091	ENSBTAG00000016683	bta:282291	http://www.kegg.jp/kegg-bin/show_pathway?bta04659+bta:282291
bta05145	Toxoplasmosis	1	15	111	8062	0.187908	0.25837	0.009009	ENSBTAG00000016683	bta:282291	http://www.kegg.jp/kegg-bin/show_pathway?bta05145+bta:282291
bta04935	Growth hormone synthesis, secretion, and action	1	15	112	8062	0.18944	0.25837	0.008929	ENSBTAG00000012007	bta:338437	http://www.kegg.jp/kegg-bin/show_pathway?bta04935+bta:338437
bta05142	Chagas disease	1	15	113	8062	0.190969	0.25837	0.00885	ENSBTAG00000016683	bta:282291	http://www.kegg.jp/kegg-bin/show_pathway?bta05142+bta:282291
bta04722	Neurotrophin signaling pathway	1	15	118	8062	0.198576	0.264768	0.008475	ENSBTAG00000016683	bta:282291	http://www.kegg.jp/kegg-bin/show_pathway?bta04722+bta:282291
bta04380	Osteoclast differentiation	1	15	123	8062	0.206115	0.270895	0.00813	ENSBTAG00000016683	bta:282291	http://www.kegg.jp/kegg-bin/show_pathway?bta04380+bta:282291
bta04926	Relaxin signaling pathway	1	15	128	8062	0.213589	0.276763	0.007813	ENSBTAG00000016683	bta:282291	http://www.kegg.jp/kegg-bin/show_pathway?bta04926+bta:282291
bta04910	Insulin signaling pathway	1	15	133	8062	0.220997	0.282385	0.007519	ENSBTAG00000012007	bta:338437	http://www.kegg.jp/kegg-bin/show_pathway?bta04910+bta:338437

bta04140	Autophagy —animal	1	15	138	8062	0.228339	0.283881	0.007246	ENSBTAG00000000163	bta:529235	http://www.kegg.jp/kegg-bin/show_pathway?bta04140+bta:529235
bta05135	Yersinia infection	1	15	138	8062	0.228339	0.283881	0.007246	ENSBTAG00000016683	bta:282291	http://www.kegg.jp/kegg-bin/show_pathway?bta05135+bta:282291
bta05162	Measles	1	15	142	8062	0.234167	0.287244	0.007042	ENSBTAG00000016683	bta:282291	http://www.kegg.jp/kegg-bin/show_pathway?bta05162+bta:282291
bta04150	mTOR signaling pathway	1	15	152	8062	0.248556	0.299874	0.006579	ENSBTAG00000000163	bta:529235	http://www.kegg.jp/kegg-bin/show_pathway?bta04150+bta:529235
bta04932	Non-alcoholic fatty liver disease	1	15	154	8062	0.251404	0.299874	0.006494	ENSBTAG00000018365	bta:282213	http://www.kegg.jp/kegg-bin/show_pathway?bta04932+bta:282213
bta05160	Hepatitis C	1	15	156	8062	0.254241	0.299874	0.00641	ENSBTAG00000016683	bta:282291	http://www.kegg.jp/kegg-bin/show_pathway?bta05160+bta:282291
bta04310	Wnt signaling pathway	1	15	159	8062	0.258478	0.301013	0.006289	ENSBTAG00000018563	bta:510821	http://www.kegg.jp/kegg-bin/show_pathway?bta04310+bta:510821
bta05161	Hepatitis B	1	15	163	8062	0.264093	0.303707	0.006135	ENSBTAG00000016683	bta:282291	http://www.kegg.jp/kegg-bin/show_pathway?bta05161+bta:282291
bta05164	Influenza A	1	15	171	8062	0.275204	0.312577	0.005848	ENSBTAG00000016683	bta:282291	http://www.kegg.jp/kegg-bin/show_pathway?bta05164+bta:282291
bta04630	JAK-STAT signaling pathway	1	15	174	8062	0.27933	0.312654	0.005747	ENSBTAG00000012007	bta:338437	http://www.kegg.jp/kegg-bin/show_pathway?bta04630+bta:338437
bta04360	Axon guidance	1	15	176	8062	0.282068	0.312654	0.005682	ENSBTAG00000044111	bta:100336601	http://www.kegg.jp/kegg-bin/show_pathway?bta04360+bta:100336601

bta01100	Metabolic pathways	4	15	1480	8062	0.291009	0.318724	0.002703	ENSBTAG00000018365丨ENSBTAG00000015505丨ENSBTAG00000019315丨ENSBTAG00000052665	bta:282213丨bta:521822丨bta:282311丨bta:507988	http://www.kegg.jp/kegg-bin/show_pathway?bta01100+bta:282213+bta:521822+bta:282311+bta:507988
bta05203	Viral carcinogenesis	1	15	200	8062	0.314181	0.340055	0.005	ENSBTAG00000016683	bta:282291	http://www.kegg.jp/kegg-bin/show_pathway?bta05203+bta:282291
bta05170	Human immunodeficiency virus 1 infection	1	15	215	8062	0.333565	0.35404	0.004651	ENSBTAG00000016683	bta:282291	http://www.kegg.jp/kegg-bin/show_pathway?bta05170+bta:282291
bta04024	cAMP signaling pathway	1	15	218	8062	0.33738	0.35404	0.004587	ENSBTAG00000016683	bta:282291	http://www.kegg.jp/kegg-bin/show_pathway?bta04024+bta:282291
bta05166	Human T-cell leukemia virus 1 infection	1	15	219	8062	0.338647	0.35404	0.004566	ENSBTAG00000016683	bta:282291	http://www.kegg.jp/kegg-bin/show_pathway?bta05166+bta:282291
bta05163	Human cytomegalovirus infection	1	15	226	8062	0.347454	0.359166	0.004425	ENSBTAG00000016683	bta:282291	http://www.kegg.jp/kegg-bin/show_pathway?bta05163+bta:282291
bta05206	MicroRNAs in cancer	1	15	248	8062	0.374425	0.382745	0.004032	ENSBTAG00000000163	bta:529235	http://www.kegg.jp/kegg-bin/show_pathway?bta05206+bta:529235
bta04060	Cytokine–cytokine receptor interaction	1	15	290	8062	0.423049	0.427698	0.003448	ENSBTAG00000037778	bta:613667	http://www.kegg.jp/kegg-bin/show_pathway?bta04060+bta:613667

bta04151	PI3K-Akt signaling pathway	1	15	350	8062	0.486422	0.486422	0.002857	ENSBTAG00000000163	bta:529235	http://www.kegg.jp/kegg-bin/show_pathway?bta04151+bta:529235

GL16 = non-castrated and slaughtered at 16 months of age; YL16 = castrated at birth and slaughtered at 16 months of age.

Table S4-1. YL16 vs. Y26 upregulated pathways (KEGG).

ID	Description	Test	Test All	Ref	Ref All	*p*-Value	FDR	Rich Factor	Gene_id	KEGG_id	URL
bta05223	Non-small-cell lung cancer	3	13	67	8062	0.000148	0.007393	0.044776	ENSBTAG00000003033 ǀENSBTAG00000016801ǀENSBTAG00000014418	bta:504939ǀbta:509500ǀbta:692183	http://www.kegg.jp/kegg-bin/show_pathway?bta05223+bta:504939+bta:509500+bta:692183
bta05222	Small-cell lung cancer	3	13	91	8062	0.000367	0.009166	0.032967	ENSBTAG00000003033ǀENSBTAG00000016801ǀENSBTAG00000014418	bta:504939ǀbta:509500ǀbta:692183	http://www.kegg.jp/kegg-bin/show_pathway?bta05222+bta:504939+bta:509500+bta:692183
bta05216	Thyroid cancer	2	13	36	8062	0.001466	0.024437	0.055556	ENSBTAG00000003033ǀENSBTAG00000016801	bta:504939ǀbta:509500	http://www.kegg.jp/kegg-bin/show_pathway?bta05216+bta:504939+bta:509500
bta00330	Arginine and proline metabolism	2	13	49	8062	0.002705	0.031418	0.040816	ENSBTAG00000052808ǀENSBTAG00000031772	bta:101904550ǀbta:508542	http://www.kegg.jp/kegg-bin/show_pathway?bta00330+bta:101904550+bta:508542
bta05202	Transcriptional misregulation in cancer	3	13	191	8062	0.003142	0.031418	0.015707	ENSBTAG00000003033ǀENSBTAG00000011266ǀENSBTAG00000016801	bta:504939ǀbta:534401ǀbta:509500	http://www.kegg.jp/kegg-bin/show_pathway?bta05202+bta:504939+bta:534401+bta:509500
bta04064	NF-kappa B signaling pathway	2	13	105	8062	0.011935	0.099457	0.019048	ENSBTAG00000003033ǀEN	bta:504939ǀbta:613667	http://www.kegg.jp/kegg-bin/show_pathway?bta04064+bta:504939+bta:613667

									SBTAG00000037778		
bta05226	Gastric cancer	2	13	149	8062	0.023158	0.165413	0.013423	ENSBTAG00000003033|ENSBTAG00000016801	bta:504939|bta:509500	http://www.kegg.jp/kegg-bin/show_pathway?bta05226+bta:504939+bta:509500
bta04950	Maturity onset diabetes of the young	1	13	26	8062	0.041153	0.24517	0.038462	ENSBTAG00000008280	bta:404168	http://www.kegg.jp/kegg-bin/show_pathway?bta04950+bta:404168
bta05200	Pathways in cancer	3	13	517	8062	0.046298	0.24517	0.005803	ENSBTAG00000003033|ENSBTAG00000011266|ENSBTAG00000016801	bta:504939|bta:534401|bta:509500	http://www.kegg.jp/kegg-bin/show_pathway?bta05200+bta:504939+bta:534401+bta:509500
bta05143	African trypanosomiasis	1	13	39	8062	0.061138	0.24517	0.025641	ENSBTAG00000037644	bta:280813	http://www.kegg.jp/kegg-bin/show_pathway?bta05143+bta:280813
bta04060	Cytokine–cytokine receptor interaction	2	13	290	8062	0.07747	0.24517	0.006897	ENSBTAG00000037778|ENSBTAG00000048508	bta:613667|bta:281867	http://www.kegg.jp/kegg-bin/show_pathway?bta04060+bta:613667+bta:281867
bta04340	Hedgehog signaling pathway	1	13	50	8062	0.077748	0.24517	0.02	ENSBTAG00000000124	bta:525833	http://www.kegg.jp/kegg-bin/show_pathway?bta04340+bta:525833
bta05144	Malaria	1	13	55	8062	0.085208	0.24517	0.018182	ENSBTAG00000037644	bta:280813	http://www.kegg.jp/kegg-bin/show_pathway?bta05144+bta:280813
bta05134	Legionellosis	1	13	56	8062	0.086693	0.24517	0.017857	ENSBTAG00000037778	bta:613667	http://www.kegg.jp/kegg-bin/show_pathway?bta05134+bta:613667

bta05213	Endometrial cancer	1	13	57	8062	0.088176	0.24517	0.017544	ENSBTAG00000003033	bta:504939	http://www.kegg.jp/kegg-bin/show_pathway?bta05213+bta:504939
bta05217	Basal cell carcinoma	1	13	62	8062	0.095558	0.24517	0.016129	ENSBTAG00000003033	bta:504939	http://www.kegg.jp/kegg-bin/show_pathway?bta05217+bta:504939
bta05221	Acute myeloid leukemia	1	13	66	8062	0.101423	0.24517	0.015152	ENSBTAG00000011266	bta:534401	http://www.kegg.jp/kegg-bin/show_pathway?bta05221+bta:534401
bta04920	Adipocytokine signaling pathway	1	13	68	8062	0.104343	0.24517	0.014706	ENSBTAG00000016801	bta:509500	http://www.kegg.jp/kegg-bin/show_pathway?bta04920+bta:509500
bta05218	Melanoma	1	13	70	8062	0.107254	0.24517	0.014286	ENSBTAG00000003033	bta:504939	http://www.kegg.jp/kegg-bin/show_pathway?bta05218+bta:504939
bta05214	Glioma	1	13	73	8062	0.111604	0.24517	0.013699	ENSBTAG00000003033	bta:504939	http://www.kegg.jp/kegg-bin/show_pathway?bta05214+bta:504939
bta05212	Pancreatic cancer	1	13	75	8062	0.114493	0.24517	0.013333	ENSBTAG00000003033	bta:504939	http://www.kegg.jp/kegg-bin/show_pathway?bta05212+bta:504939
bta05220	Chronic myeloid leukemia	1	13	76	8062	0.115934	0.24517	0.013158	ENSBTAG00000003033	bta:504939	http://www.kegg.jp/kegg-bin/show_pathway?bta05220+bta:504939
bta03320	PPAR signaling pathway	1	13	79	8062	0.120245	0.24517	0.012658	ENSBTAG00000016801	bta:509500	http://www.kegg.jp/kegg-bin/show_pathway?bta03320+bta:509500
bta04115	p53 signaling pathway	1	13	79	8062	0.120245	0.24517	0.012658	ENSBTAG00000003033	bta:504939	http://www.kegg.jp/kegg-bin/show_pathway?bta04115+bta:504939
bta05210	Colorectal cancer	1	13	87	8062	0.131646	0.24517	0.011494	ENSBTAG00000003033	bta:504939	http://www.kegg.jp/kegg-bin/show_pathway?bta05210+bta:504939

bta04350	TGF-beta signaling pathway	1	13	90	8062	0.135886	0.24517	0.011111	ENSBTAG00000048508	bta:281867	http://www.kegg.jp/kegg-bin/show_pathway?bta04350+bta:281867
bta04657	IL-17 signaling pathway	1	13	90	8062	0.135886	0.24517	0.011111	ENSBTAG00000037778	bta:613667	http://www.kegg.jp/kegg-bin/show_pathway?bta04657+bta:613667
bta04061	Viral protein interaction with cytokine and cytokine receptor	1	13	91	8062	0.137295	0.24517	0.010989	ENSBTAG00000037778	bta:613667	http://www.kegg.jp/kegg-bin/show_pathway?bta04061+bta:613667
bta05323	Rheumatoid arthritis	1	13	97	8062	0.145706	0.251216	0.010309	ENSBTAG00000037778	bta:613667	http://www.kegg.jp/kegg-bin/show_pathway?bta05323+bta:613667
bta04928	Parathyroid hormone synthesis, secretion, and action	1	13	105	8062	0.156801	0.252567	0.009524	ENSBTAG00000016801	bta:509500	http://www.kegg.jp/kegg-bin/show_pathway?bta04928+bta:509500
bta05146	Amoebiasis	1	13	106	8062	0.158179	0.252567	0.009434	ENSBTAG00000037778	bta:613667	http://www.kegg.jp/kegg-bin/show_pathway?bta05146+bta:613667
bta04659	Th17 cell differentiation	1	13	110	8062	0.163669	0.252567	0.009091	ENSBTAG00000016801	bta:509500	http://www.kegg.jp/kegg-bin/show_pathway?bta04659+bta:509500
bta04919	Thyroid hormone signaling pathway	1	13	118	8062	0.174549	0.252567	0.008475	ENSBTAG00000016801	bta:509500	http://www.kegg.jp/kegg-bin/show_pathway?bta04919+bta:509500
bta04668	TNF signaling pathway	1	13	119	8062	0.1759	0.252567	0.008403	ENSBTAG00000037778	bta:613667	http://www.kegg.jp/kegg-bin/show_pathway?bta04668+bta:613667

bta04110	Cell cycle	1	13	121	8062	0.178595	0.252567	0.008264	ENSBTAG00000003033	bta:504939	http://www.kegg.jp/kegg-bin/show_pathway?bta04110+bta:504939
bta04068	FoxO signaling pathway	1	13	127	8062	0.186633	0.252567	0.007874	ENSBTAG00000003033	bta:504939	http://www.kegg.jp/kegg-bin/show_pathway?bta04068+bta:504939
bta04915	Estrogen signaling pathway	1	13	130	8062	0.190625	0.252567	0.007692	ENSBTAG00000047502	bta:535704	http://www.kegg.jp/kegg-bin/show_pathway?bta04915+bta:535704
bta00230	Purine metabolism	1	13	131	8062	0.191951	0.252567	0.007634	ENSBTAG00000014418	bta:692183	http://www.kegg.jp/kegg-bin/show_pathway?bta00230+bta:692183
bta04550	Signaling pathways regulating pluripotency of stem cells	1	13	138	8062	0.201181	0.25475	0.007246	ENSBTAG00000048508	bta:281867	http://www.kegg.jp/kegg-bin/show_pathway?bta04550+bta:281867
bta04210	Apoptosis	1	13	140	8062	0.2038	0.25475	0.007143	ENSBTAG00000003033	bta:504939	http://www.kegg.jp/kegg-bin/show_pathway?bta04210+bta:504939
bta05224	Breast cancer	1	13	146	8062	0.21161	0.258061	0.006849	ENSBTAG00000003033	bta:504939	http://www.kegg.jp/kegg-bin/show_pathway?bta05224+bta:504939
bta04218	Cellular senescence	1	13	151	8062	0.218064	0.2596	0.006623	ENSBTAG00000003033	bta:504939	http://www.kegg.jp/kegg-bin/show_pathway?bta04218+bta:504939
bta05225	Hepatocellular carcinoma	1	13	170	8062	0.242147	0.279416	0.005882	ENSBTAG00000003033	bta:504939	http://www.kegg.jp/kegg-bin/show_pathway?bta05225+bta:504939
bta04621	NOD-like receptor signaling pathway	1	13	173	8062	0.245886	0.279416	0.00578	ENSBTAG00000037778	bta:613667	http://www.kegg.jp/kegg-bin/show_pathway?bta04621+bta:613667

bta04062	Chemokine signaling pathway	1	13	183	8062	0.258228	0.28692	0.005464	ENSBTAG00000037778	bta:613667	http://www.kegg.jp/kegg-bin/show_pathway?bta04062+bta:613667
bta05167	Kaposi's sarcoma-associated herpesvirus infection	1	13	193	8062	0.270384	0.293895	0.005181	ENSBTAG00000037778	bta:613667	http://www.kegg.jp/kegg-bin/show_pathway?bta05167+bta:613667
bta05169	Epstein–Barr virus infection	1	13	212	8062	0.292974	0.311674	0.004717	ENSBTAG00000003033	bta:504939	http://www.kegg.jp/kegg-bin/show_pathway?bta05169+bta:504939
bta05206	MicroRNAs in cancer	1	13	248	8062	0.334015	0.347932	0.004032	ENSBTAG00000009983	bta:515748	http://www.kegg.jp/kegg-bin/show_pathway?bta05206+bta:515748
bta04010	MAPK signaling pathway	1	13	287	8062	0.375987	0.38366	0.003484	ENSBTAG00000003033	bta:504939	http://www.kegg.jp/kegg-bin/show_pathway?bta04010+bta:504939
bta01100	Metabolic pathways	3	13	1480	8062	0.436806	0.436806	0.002027	ENSBTAG00000052808丨ENSBTAG00000031772丨ENSBTAG00000014418	bta:101904550丨bta:508542丨bta:692183	http://www.kegg.jp/kegg-bin/show_pathway?bta01100+bta:101904550+bta:508542+bta:692183

YL16 = castrated at birth and slaughtered at 16 months of age; YL26 = castrated at birth and slaughtered at 26 months of age.

Table S4-2. YL16 vs. Y26 downregulated pathways (KEGG)

ID	Description	Test	Test All	Ref	Ref All	*p*-Value	FDR	Rich Factor	Gene_id	KEGG_id	URL
bta00561	Glycerolipid metabolism	2	8	60	8062	0.001482	0.028156	0.033333	ENSBTAG00000008160丨ENSBTAG00000001582	bta:785489丨bta:525193	http://www.kegg.jp/kegg-bin/show_pathway?bta00561+bta:785489+bta:525193
bta00072	Synthesis and degradation of ketone bodies	1	8	9	8062	0.0089	0.073894	0.111111	ENSBTAG00000011839	bta:407767	http://www.kegg.jp/kegg-bin/show_pathway?bta00072+bta:407767
bta00900	Terpenoid backbone biosynthesis	1	8	21	8062	0.020658	0.073894	0.047619	ENSBTAG00000011839	bta:407767	http://www.kegg.jp/kegg-bin/show_pathway?bta00900+bta:407767
bta04614	Renin–angiotensin system	1	8	25	8062	0.024551	0.073894	0.04	ENSBTAG00000034402	bta:509235	http://www.kegg.jp/kegg-bin/show_pathway?bta04614+bta:509235
bta00650	Butanoate metabolism	1	8	26	8062	0.025522	0.073894	0.038462	ENSBTAG00000011839	bta:407767	http://www.kegg.jp/kegg-bin/show_pathway?bta00650+bta:407767
bta00040	Pentose and glucuronate interconversions	1	8	29	8062	0.028429	0.073894	0.034483	ENSBTAG00000001582	bta:525193	http://www.kegg.jp/kegg-bin/show_pathway?bta00040+bta:525193
bta00052	Galactose metabolism	1	8	29	8062	0.028429	0.073894	0.034483	ENSBTAG00000001582	bta:525193	http://www.kegg.jp/kegg-bin/show_pathway?bta00052+bta:525193
bta00051	Fructose and mannose metabolism	1	8	33	8062	0.032295	0.073894	0.030303	ENSBTAG00000001582	bta:525193	http://www.kegg.jp/kegg-bin/show_pathway?bta00051+bta:525193

bta00790	Folate biosynthesis	1	8	37	8062	0.036147	0.073894	0.027027	ENSBTAG00000001582	bta:525193	http://www.kegg.jp/kegg-bin/show_pathway?bta00790+bta:525193
bta01100	Metabolic pathways	4	8	1480	8062	0.042354	0.073894	0.002703	ENSBTAG00000008160 | ENSBTAG00000011839 | ENSBTAG00000001582 | ENSBTAG00000021118	bta:785489 | bta:407767 | bta:525193 | bta:539047	http://www.kegg.jp/kegg-bin/show_pathway?bta01100+bta:785489+bta:407767+bta:525193+bta:539047
bta00970	Aminoacyl-tRNA biosynthesis	1	8	44	8062	0.042855	0.073894	0.022727	ENSBTAG00000018072	bta:505390	http://www.kegg.jp/kegg-bin/show_pathway?bta00970+bta:505390
bta00280	Valine, leucine, and isoleucine degradation	1	8	48	8062	0.04667	0.073894	0.020833	ENSBTAG00000011839	bta:407767	http://www.kegg.jp/kegg-bin/show_pathway?bta00280+bta:407767
bta00830	Retinol metabolism	1	8	63	8062	0.060858	0.088946	0.015873	ENSBTAG00000021118	bta:539047	http://www.kegg.jp/kegg-bin/show_pathway?bta00830+bta:539047
bta04612	Antigen processing and presentation	1	8	73	8062	0.070214	0.09529	0.013699	ENSBTAG00000046979	bta:281281	http://www.kegg.jp/kegg-bin/show_pathway?bta04612+bta:281281
bta03320	PPAR signaling pathway	1	8	79	8062	0.075788	0.095998	0.012658	ENSBTAG00000011839	bta:407767	http://www.kegg.jp/kegg-bin/show_pathway?bta03320+bta:407767
bta00564	Glycerophospholipid metabolism	1	8	95	8062	0.090509	0.10748	0.010526	ENSBTAG00000008160	bta:785489	http://www.kegg.jp/kegg-bin/show_pathway?bta00564+bta:785489
bta04974	Protein digestion and absorption	1	8	108	8062	0.102319	0.114357	0.009259	ENSBTAG00000034402	bta:509235	http://www.kegg.jp/kegg-bin/show_pathway?bta04974+bta:509235

bta04142	Lysosome	1	8	129	8062	0.121114	0.127842	0.007752	ENSBTAG00000046979	bta:281281	http://www.kegg.jp/kegg-bin/show_pathway?bta04142+bta:281281
bta04621	NOD-like receptor signaling pathway	1	8	173	8062	0.159379	0.159379	0.00578	ENSBTAG00000038938	bta:781710	http://www.kegg.jp/kegg-bin/show_pathway?bta04621+bta:781710

YL16 = castrated at birth and slaughtered at 16 months of age; YL26 = castrated at birth and slaughtered at 26 months of age.

Table S5. Summary of the mode fitness of the UHPLC-QTOF/MS analysis of the comparisons among the three groups.

Type	R2X(cum)	R2Y(cum)	Q2(cum)	Title
Positive				
PCA	0.542	—	—	QC
PCA	0.585	—	—	YL16_vs_GL16
OPLS-DA	0.33	0.987	0.707	YL16_vs_GL16
OPLS-DA permutation	—	—	-0.075	YL16_vs_GL16
PCA	0.567	—	—	YL26_vs_GL16
OPLS-DA	0.284	0.995	0.76	YL26_vs_GL16
OPLS-DA permutation	—	—	0.024	YL26_vs_GL16
PCA	0.541	—	—	YL26_vs_YL16
OPLS-DA	0.256	0.99	0.339	YL26_vs_YL16
OPLS-DA permutation	—	—	0.052	YL26_vs_YL16
Negative				
PCA	0.505	—	—	QC
PCA	0.556	—	—	YL16_vs_GL16
OPLS-DA	0.523	0.995	0.731	YL16_vs_GL16
OPLS-DA permutation	—	—	-0.038	YL16_vs_GL16
PCA	0.518	—	—	YL26_vs_GL16
OPLS-DA	0.48	0.996	0.677	YL26_vs_GL16
OPLS-DA permutation	—	—	0.053	YL26_vs_GL16
PCA	0.594	—	—	YL26_vs_YL16
OPLS-DA	0.237	0.984	0.211	YL26_vs_YL16
OPLS-DA permutation	—	—	0.075	YL26_vs_YL16

GL16 = non-castrated and slaughtered at 16 months of age; YL16 = castrated at birth and slaughtered at 16 months of age; YL26 = castrated at birth and slaughtered at 26 months of age.

Table S6. Identification of different metabolites from any two groups of GL16, YL16, and YL26 in liver.

Name	Description	VIP	Fold change	*p*-Value
	GL16 vs. YL16			
	Positive mode			
M252T118	5'-Deoxyadenosine	1.014891	1.3705433	0.021283242
M385T378_1	S-adenosyl-L-homocysteine	1.171193	1.508589	0.046733382
M120T256_2	Tyramine	10.423597	0.7342128	0.030981084
M102T396_2	1-Aminocyclopropanecarboxylic acid	2.1790063	0.8553612	0.013394494
M104T371	4-Aminobutyric acid	1.6028921	0.4497935	0.000831034
M219T406	5-L-glutamyl-L-alanine	7.2405952	3.3222118	0.000383226
M118T274_2	Betaine	16.973297	1.6928356	0.003169148
M114T172_2	Creatinine	5.2999432	0.8704624	0.003709604
M251T389	gamma-Glutamylcysteine	1.559874	1.6575798	0.029298445
M277T459	gamma-L-glutamyl-L-glutamic acid	6.5950523	1.2137029	0.03992313
M308T395	Glutathione	1.3714943	2.1178736	0.046121839
M176T403	Guanidinosuccinic acid	1.3898325	0.5278148	0.082781267
M90T406	L-alanine	1.6485567	2.5511504	0.000464988
M134T401_2	L-aspartate	1.368293	0.8696667	0.082219207
M241T370	L-cystine	1.2937633	0.6210038	0.021357622
M132T341	L-leucine	4.102839	0.626537	0.02437289
M150T282_2	L-methionine	2.9671706	0.7441544	0.024677777
M166T256_2	L-phenylalanine	6.8557525	0.7589614	0.063161257
M147T371_2	L-pyroglutamic acid	5.1679845	1.6265847	0.033353376
M106T374	L-serine	1.5666781	0.7105684	0.002145861
M203T500	NG,NG-dimethyl-L-arginine (ADMA)	12.00918	0.6844221	0.001872284
M133T512	Ornithine	2.5497641	0.6683781	0.058234456
M147T299	4-Hydroxycinnamic acid	1.2930777	0.8059884	0.001256982
M204T304	Acetylcarnitine	2.4318793	1.6616149	0.042069856
M428T170	Stearoylcarnitine	1.81717	0.7085511	0.028699204
M855T138	1-Stearoyl-2-oleoyl-sn-glycerol 3-phosphocholine (SOPC)	1.0193219	1.5335331	0.000603664
M173T430	Glycerol 3-phosphate	4.0430306	1.5082548	0.068458484
M258T430_2	Glycerophosphocholine	6.6868862	1.1937897	0.031561401
M779T146	PC (16:0/16:0)	1.0879923	1.3873234	0.062597251
M87T372	DL-3-hydroxybutyric acid	1.7459883	0.5665186	0.001200358
M137T220	Hypoxanthine	4.5009401	1.36661	0.030118629
M153T221_2	Xanthine	3.9091135	0.8227115	0.004829249
M192T259	5-Hydroxyindoleacetate	1.4103189	0.6477859	0.029000901
M61T105_2	Urea	1.7166403	0.8302776	0.020968564
M104T324	Choline	3.1241936	0.7479065	0.069072028
M70T309	Diethanolamine	3.2619638	0.7691175	0.003167973
M112T391	Histamine	3.4240688	0.3231744	0.039471313
M300T93	Sphingosine	2.0720666	0.6554054	0.003349835
M786T396	Flavin adenine dinucleotide (FAD)	1.3352761	1.1991535	0.007013644
M360T391	Isomaltose	3.9090883	0.6263494	0.049874867
M209T259	L-kynurenine	1.2166517	0.6785907	0.04287285
M522T450	Maltotriose	3.8015785	0.4900111	0.092844328
M204T278	N-acetylmannosamine	1.3566425	1.4632753	0.04711912
M220T276_2	Pantothenate	3.3755962	0.6806216	0.003025035
M241T427	L-anserine	1.0383936	1.5935981	0.062059608
M136T299_2	Dopamine	3.002207	0.8032562	0.000491583
M152T110	Norepinephrine	2.2014723	0.5956669	0.001955925

M131T256	DL-3-phenyllactic acid	1.3371268	0.7252555	0.027834439
M377T215	Riboflavin	1.5609748	1.2858602	0.027173348
M298T198	1-methylguanosine	1.2034636	0.6232983	0.015180942
M269T220	Inosine	1.3489559	1.3924456	0.028959042
M282T283	N6-methyladenosine	2.6656356	0.6510591	0.001206437
M184T37	4-Pyridoxic acid	1.1099014	0.7594808	0.051498611
M123T456	Nicotinamide	6.3225709	2.1652033	0.03926382
M226T182	Cytidine	1.1726078	0.5301414	0.055459711
M348T403_1	Adenosine 3'-monophosphate	1.7839491	0.7327516	0.072092821
M426T228	Cholic acid	1.607527	0.583122	0.070899961
M498T106	Taurocholate	1.5636043	0.322213	0.033309741
M482T135	Tauroursodeoxycholic acid	3.3496952	0.5214364	0.068929551
M359T106	(-)-Riboflavin	1.1454448	0.7676781	0.096176174
M130T69	.beta.-Homoproline	1.2874727	1.5695226	0.06657048
M809T40_1	1,2-dioleoyl-sn-glycero-3-phosphatidylcholine	12.660038	1.3096384	0.012512702
M719T150	1-Palmitoyl-2-oleoyl-sn-glycero-3-phosphoethanolamine	1.8649375	1.2526624	0.017779074
M538T189	1-Palmitoyllysophosphatidylcholine	1.4731882	0.7193845	0.074926238
M482T196	1-Stearoyl-2-hydroxy-sn-glycero-3-phosphoethanolamine	6.6006546	0.7014271	0.001850363
M325T390	3.alpha.-Mannobiose	4.3879606	0.5820246	0.035979972
M240T384	Ala-Ser	1.2354765	1.2578255	0.005258554
M245T340	Arg-Ala	1.3184488	0.8150817	0.045221405
M179T371	D-glucono-1,5-lactone	1.1655956	0.6703917	0.029486618
M101T371	DL-2,4-diaminobutyric acid	1.1221541	1.5939368	0.017327861
M188T256	DL-Indole-3-lactic acid	3.3885187	0.7716862	0.027093027
M147T523	D-pipecolinic acid	1.9213477	0.832927	0.060373856
M295T345	gamma-L-glutamyl-L-phenylalanine	1.2972721	0.7456081	0.002334547
M133T374	L-asparagine	1.8266608	0.7757058	0.000839134
M182T299_2	L-tyrosine	3.9190182	0.7850881	0.000845838
M337T192	MG (18:2(9Z,12Z)/0:0/0:0)[rac]	1.8502549	0.5527238	0.000405531
M146T257	Oxyquinoline	1.3883361	0.7406954	0.005055282
M213T429	Pro-Asp	1.9243099	0.225371	0.043757656
M170T110	Pyridoxine	3.0314425	0.5948402	0.001460726
M759T119	Thioetheramide-PC	20.65494	0.6399673	0.030223579
M165T299_2	trans-2-Hydroxycinnamic acid	3.5980026	0.7803033	0.000296119
	Negative mode			
M136T103	Anthranilic acid (Vitamin L1)	1.6484694	0.6291142	0.015155202
M217T407	5-L-glutamyl-L-alanine	2.050598	2.6530467	0.001298711
M132T401	D-aspartic acid	1.7595563	0.845093	0.072556041
M114T310_2	D-proline	2.132916	0.7419424	0.004166792
M275T459_2	gamma-L-glutamyl-L-glutamic acid	2.8286999	1.3117458	0.022832018
M74T348	Glycine	1.0537534	0.7062122	0.004169157
M146T396_2	L-glutamate	6.090233	0.8207861	0.003051202
M145T428	L-glutamine	1.1966412	2.6290576	1.23874E-05
M130T274_2	L-leucine	7.1249543	0.6916607	0.000141954
M164T256_2	L-phenylalanine	4.466947	0.6966713	0.036075193
M104T374	L-serine	1.2093659	0.7564507	0.004160114
M116T299_2	L-valine	2.426006	0.7350488	0.000815259
M271T80_2	16-Hydroxypalmitic acid	4.4014279	1.664933	0.004179182
M279T46	Linoleic acid	25.264051	0.7260491	0.006304864
M227T50	Myristic acid	14.727234	1.4614469	0.025945439
M225T50	Myristoleic acid	6.14448	1.3104261	0.070874651
M281T153	Oleic acid	1.0486785	0.7920324	0.073273387
M171T454	Glycerol 3-phosphate	1.380496	1.340201	0.093102127
M129T371	(S)-2-hydroxyglutarate	1.3235918	0.7567745	0.090626506
M197T52	3-Hydroxydodecanoic acid	1.2727473	0.6880676	0.00153321

M391T371	1,3-Dimethyluric acid	1.506117	0.3198681	0.029441503
M151T220	Xanthine	2.5336681	0.8913954	0.028331969
M203T257	L-tryptophan	1.6563214	0.7230387	0.013300128
M141T286	2-Oxoadipic acid	2.0658995	1.4154846	0.05735099
M129T60	Dihydroxyfumarate	3.2420149	0.4436134	0.006997189
M177T114	L-gulonic gamma-lactone	9.3241038	1.5304141	0.013443022
M124T469	Ciliatine	1.3997758	0.7114495	0.030268028
M124T296_2	Taurine	6.6832719	0.8001867	0.01997105
M195T397	D-gluconate	5.1124001	0.74999	0.054560059
M341T391	D-maltose	4.1946827	0.5767159	0.018928796
M179T267	D-mannose	3.0224275	0.6997936	0.000404443
M161T391	D-Tagatose	2.5118921	0.6074267	0.045957238
M181T349	D-threitol	1.3102323	0.2182198	0.031075564
M401T398	Galactinol	1.0852969	0.7537984	0.007536862
M218T275_2	Pantothenate	5.6402692	0.6290092	0.000510558
M211T349	Xylitol	1.4259201	0.7729866	0.048353127
M267T222	Inosine	2.4446054	1.320196	0.022940332
M283T312	Xanthosine	1.0346495	1.4827766	0.002314482
M182T42	4-Pyridoxic acid	2.6096134	0.75136	0.044484009
M346T404	Adenosine 3'-monophosphate	1.051078	0.7275805	0.051995304
M407T228	Cholic acid	1.2831385	0.5592853	0.045128557
M391T159	Deoxycholic acid	1.0453416	0.6176209	0.044196201
M998T83	Taurochenodeoxycholate	1.0135206	1.7324524	0.051512312
M514T177	Taurocholate	2.1242716	0.5026191	0.014766875
M447T124	1,3,5(10)-Estratrien-3,17.beta.-diol 17-glucosiduronate	1.6267989	5.0446742	0.0009396
M147T113	D-arabinono-1,4-lactone	1.2927187	0.7785823	0.056665367
M277T367	gamma-Glutamyl-L-methionine	1.3395817	1.6168987	0.006498159
M131T344	L-asparagine	1.001999	0.485121	0.004300778
M118T350	L-threonine	2.393192	0.6822075	0.006671592
M355T177_2	PGF1a	1.3295867	1.7210257	0.007075391
	YL16 vs. YL26			
	Positive mode			
M150T155	Triethanolamine	1.0228119	1.1798519	0.08934632
M120T256_2	Tyramine	15.77549	1.5262428	0.004043331
M219T406	5-L-glutamyl-L-alanine	6.997439	0.5191533	0.010297325
M223T452	Allocystathionine	1.0091424	1.3587636	0.006446345
M118T300	Betaine	3.3320944	1.2230059	0.000913333
M114T172_2	Creatinine	6.0128776	1.1590291	0.001596286
M166T364	DL-methionine sulfoxide	2.566289	2.2707019	0.016277116
M331T255	DL-phenylalanine	1.1029037	1.9502606	0.005798048
M116T337	D-proline	2.3348929	2.315633	0.004234928
M277T459	gamma-L-glutamyl-L-glutamic acid	9.5881508	0.7679767	0.014964554
M176T403	Guanidinosuccinic acid	1.4486024	0.619922	0.016315762
M90T406	L-alanine	1.6381965	0.5764054	0.009811057
M175T577	L-arginine	4.8211275	0.5168495	0.026098457
M148T459	L-glutamate	3.5663172	0.7751857	0.017975494
M156T581	L-histidine	3.4921963	0.6326643	0.032170985
M132T262_2	L-leucine	6.6843207	1.2593131	0.001569863
M150T282_2	L-methionine	3.2609552	1.3081942	0.009375285
M166T256_2	L-phenylalanine	11.18569	1.521839	0.004455352
M116T553	L-proline	1.0544553	1.3316839	0.064736863
M147T429	L-pyroglutamic acid	4.6393388	0.7449064	0.05855504
M106T374	L-serine	1.4246928	1.2733458	0.009074161
M161T531	N6-methyl-L-lysine	1.5806473	0.840358	0.073716257
M203T500	NG,NG-dimethyl-L-arginine (ADMA)	7.0246565	1.2065366	0.052284374
M133T512	Ornithine	3.4155502	1.6379183	0.023131309
M298T95	Linoleic acid	1.3258557	1.5414864	0.093370714

M833T90	1-Stearoyl-2-oleoyl-sn-glycerol 3-phosphocholine (SOPC)	2.1327119	0.7687555	0.013629212
M173T430	Glycerol 3-phosphate	4.6067452	0.7319575	0.063344714
M189T94	7-Methylxanthine	1.0776609	1.4899838	0.086616224
M132T159_3	3-Methylindole	1.2642753	1.5503779	0.04045034
M192T259	5-Hydroxyindoleacetate	2.1424612	1.7322576	0.014237813
M158T54	Indoleacetic acid	1.0971243	1.519491	0.004838617
M126T469	Ciliatine	3.0512064	0.6946882	0.068217265
M102T293	Betaine aldehyde	1.5863667	0.7112654	0.099899497
M70T309	Diethanolamine	3.8356946	1.3227558	0.001774474
M300T93	Sphingosine	1.5230505	1.3427037	0.070883637
M209T259	L-kynurenine	1.9058035	1.6754351	0.016790197
M667T489	Maltotetraose	1.8539173	0.4275221	0.058109354
M220T276_2	Pantothenate	2.0436033	1.2471258	0.079159738
M684T489	Stachyose	2.1983891	0.4266437	0.057212098
M241T427	L-anserine	1.5818301	0.5798307	0.035836699
M227T417	L-carnosine	10.744485	0.6603245	0.04396741
M136T299_2	Dopamine	1.7234513	1.1555764	0.044175573
M131T256	DL-3-phenyllactic acid	1.9443149	1.5102226	0.003657047
M268T173	Adenosine	2.931743	1.2790416	0.032413396
M428T487_1	Adenosine 3',5'-diphosphate (PAP)	1.076481	1.5801434	0.033825637
M123T456	Nicotinamide	7.5466794	0.4958621	0.062206598
M226T182	Cytidine	1.0369137	1.4871072	0.092753521
M348T403_1	Adenosine 3'-monophosphate	4.6191205	1.8849906	0.000146184
M359T106	(-)-Riboflavin	1.1214798	1.2300411	0.079610802
M130T361	.beta.-Homoproline	1.0702781	0.7135134	0.024132192
M136T248	Adenine	3.4857549	0.2026978	0.046816939
M245T367	Arg-Ala	1.1444015	1.8381989	0.002176318
M304T443	Arg-Glu	1.1651065	1.3315132	0.017572284
M168T158	D-(-)-lyxose	1.254449	1.4489566	0.010456432
M188T256	DL-indole-3-lactic acid	3.2793413	1.2453595	0.03609407
M147T523	D-pipecolinic acid	2.4184047	1.2477062	0.073248417
M295T345	gamma-L-glutamyl-L-phenylalanine	1.7150017	1.4363772	0.00118896
M269T295	His-Ile	1.2190044	1.3777227	0.015340595
M243T358_2	His-Ser	1.4037495	1.5858316	0.00078744
M257T342	His-Thr	1.0254844	1.4110722	0.010156257
M261T353	Ile-Glu	1.055556	1.1989487	0.01845955
M133T374	L-asparagine	2.0398334	1.2857005	0.001021514
M274T88	Leu-Thr	1.15486	1.5320173	0.032913186
M182T299_2	L-tyrosine	2.0562678	1.1515881	0.034293708
M243T60	Lumichrome	2.1375944	1.4165378	0.021688139
M198T417	N-acetyl-L-histidine	1.2652438	0.6974061	0.006934177
M229T160	Norharmane	1.0989632	2.9401133	0.029725649
M146T257	Oxyquinoline	1.6486157	1.3656575	0.003606254
M223T233	Phe-Gly	1.2531917	1.7441608	0.051713293
M198T448	sn-Glycerol 3-phosphoethanolamine	1.6671314	1.398378	0.018619817
M759T60	Thioetheramide-PC	36.980131	1.4419323	0.01043254
M165T299_2	trans-2-Hydroxycinnamic acid	1.702808	1.1511287	0.057139073
	Negative mode			
M217T407	5-L-glutamyl-L-alanine	2.1215212	0.6288043	0.042304653
M114T310_2	D-proline	2.9764313	1.3111577	0.002800128
M275T459_2	gamma-L-glutamyl-L-glutamic acid	4.1722202	0.7046314	0.007698953
M183T262	L-glutamine	2.9747526	2.5913366	0.042457257
M154T440	L-histidine	1.7501883	0.6826453	0.017218962
M130T274_2	L-leucine	8.5191192	1.3258511	0.001537901
M164T256_2	L-phenylalanine	10.36919	1.5899563	0.003726925
M104T374	L-serine	1.8917974	1.2476082	0.027995708

M116T299_2	L-valine	3.474998	1.3070702	0.000211671
M115T405_2	Maleic acid	4.207806	0.8521327	0.055835621
M113T374	Dihydrouracil	1.04374	1.2029283	0.010544772
M271T64	16-Hydroxypalmitic acid	5.2284952	0.6364133	3.23696E-05
M161T373	3-Hydorxy-3-methylglutaric acid	1.951218	0.7616239	0.006079131
M436T210	L-palmitoylcarnitine	1.0776092	0.6309739	0.019714427
M281T136	Oleic acid	2.4347585	1.2710775	0.065919709
M511T47	Palmitic acid	4.1133962	0.6639564	0.025697113
M171T431	Glycerol 3-phosphate	6.2937191	0.7207233	0.034678197
M147T392	(S)-2-hydroxyglutarate	1.9960797	0.7504052	0.012979991
M197T52	3-Hydroxydodecanoic acid	1.3578726	1.3874097	0.073037199
M133T405_2	L-malic acid	11.759111	0.8023539	0.023200567
M151T249	Xanthine	7.3748875	3.1048876	0.013104185
M190T260	5-Hydroxyindoleacetate	1.0607117	1.6583111	0.026235139
M203T257	L-tryptophan	2.1876088	1.29618	0.016822252
M141T316	2-Oxoadipic acid	2.2800739	1.2493732	0.038506061
M129T110	Dihydroxyfumarate	4.7428549	1.6995904	0.012340298
M177T158	L-gulonic gamma-lactone	3.7601157	1.1687351	0.05615483
M71T376	Dihydroxyacetone	1.540578	0.790621	0.015690893
M229T402	Dihydroxyacetone phosphate	1.5876597	1.682929	3.86425E-05
M299T158	D-ribose	2.219275	1.6810031	0.01974793
M289T462	D-ribose 5-phosphate	1.0191843	0.6443514	0.010662427
M259T71	Fructose 1-phosphate	1.1784268	1.9580057	0.069388062
M401T398	Galactinol	1.2870248	1.1529966	0.096007998
M181T297_2	L-iditol	4.8880159	1.2825666	0.067084683
M218T275_2	Pantothenate	4.06082	1.2998244	0.074396061
M143T306	Pyruvaldehyde	2.7011374	1.1229385	0.060992176
M225T417	L-carnosine	4.3012272	0.6865717	0.059415383
M283T312	Xanthosine	1.2333147	0.7277197	0.009696864
M346T428	Adenosine 3'-monophosphate	1.4571678	1.4685157	0.000502353
M407T228	Cholic acid	2.3231559	1.9902592	0.081524285
M482T76	Taurolithocholic acid	8.3393149	1.5843316	0.086192385
M295T61	9(S)-HODE	2.3200956	1.2607305	0.067824412
M149T158	D-lyxose	9.7423097	1.4350871	0.014144069
M131T374	L-asparagine	2.003715	1.226124	0.005274204
M241T64	Lumichrome	3.1050455	1.2346368	0.077139615
M310T268	Muramic acid	1.3474298	0.6287282	0.04411085
M196T448	sn-Glycerol 3-phosphoethanolamine	1.1657587	1.3710678	0.01388203
	GL16 vs. YL26			
	Positive mode			
M298T102	S-methyl-5'-thioadenosine	3.6078072	1.403362	0.083016848
M102T396_2	1-Aminocyclopropanecarboxylic acid	3.355057	0.8292743	0.002401486
M104T371	4-Aminobutyric acid	1.5895038	0.5069019	0.001870752
M219T406	5-L-glutamyl-L-alanine	3.3816014	1.7247373	0.052620117
M118T274_2	Betaine	16.747102	1.7346391	0.000256384
M132T347_2	Creatine	8.4626325	1.1418547	0.09888182
M116T337	D-proline	1.1334448	1.4681466	0.08223927
M176T403	Guanidinosuccinic acid	2.0155595	0.327204	0.01853136
M134T401_2	L-aspartate	2.379875	0.8557965	0.01668204
M241T353	L-cystine	1.1535125	0.8111659	0.080210593
M148T396_2	L-glutamate	8.6441643	0.8197888	0.003028125
M156T443	L-histidine	5.6519256	0.6301161	0.00373493
M132T341	L-leucine	5.8177475	0.5396096	0.010333166
M166T256_2	L-phenylalanine	4.1769427	1.1550171	0.064410319
M147T371_2	L-pyroglutamic acid	7.053545	2.0432547	0.025847819
M161T531	N6-methyl-L-lysine	1.8367028	0.7283643	0.01867182
M203T500	NG,NG-dimethyl-L-arginine (ADMA)	10.044594	0.8257803	0.004512063

M147T299	4-Hydroxycinnamic acid	1.010121	0.9110117	0.065322493
M113T89	Uracil	1.7715963	0.7184612	0.083531622
M204T304	Acetylcarnitine	1.6083286	1.4384004	0.074604935
M428T170	Stearoylcarnitine	1.9992192	0.7309876	0.04682286
M811T110	1-Stearoyl-2-oleoyl-sn-glycerol 3-phosphocholine (SOPC)	12.96676	1.5701102	0.013740603
M258T454	Glycerophosphocholine	4.961611	1.3810777	0.085891033
M779T146	PC (16:0/16:0)	1.1525465	1.3843905	0.004122285
M87T372	DL-3-hydroxybutyric acid	1.6925535	0.6449593	0.004069752
M153T221_2	Xanthine	3.821642	0.8761449	0.064464357
M126T469	Ciliatine	3.4908515	0.5501926	0.011792304
M104T384_2	Choline	4.6206365	1.0816407	0.058064252
M184T470_2	Phosphorylcholine	26.755171	1.2235425	0.017194402
M360T391	Isomaltose	4.776859	0.6183138	0.0926764
M667T489	Maltotetraose	2.3692358	0.2359965	0.008038488
M522T450	Maltotriose	5.245626	0.3236244	0.026132687
M204T278	N-acetylmannosamine	1.4422152	1.5081459	0.00197195
M310T373	N-acetylneuraminic acid	1.1043033	0.7344716	0.023804317
M220T276_2	Pantothenate	2.4384355	0.8488208	0.045001956
M684T489	Stachyose	2.8136128	0.2354013	0.007155475
M227T417	L-carnosine	7.4685413	0.7061044	0.064834326
M152T110	Norepinephrine	2.072911	0.7007412	0.015786979
M298T198	1-methylguanosine	1.0089465	0.7554763	0.056500258
M282T283	N6-methyladenosine	2.4017898	0.7463341	0.014207725
M428T487_1	Adenosine 3',5'-diphosphate (PAP)	1.2178904	2.2003562	0.005130315
M124T222	Nicotinate	1.0964024	0.68081	0.003377346
M348T403_1	Adenosine 3'-monophosphate	2.5405982	1.3812299	0.027152602
M482T152_2	Taurochenodeoxycholate	5.0140257	0.5854138	0.091120449
M498T106	Taurocholate	1.623568	0.4203055	0.055881935
M500T152_2	Taurodeoxycholic acid	11.849219	0.5741549	0.099002321
M482T135	Tauroursodeoxycholic acid	4.0687817	0.516366	0.029734612
M146T375_2	(3-Carboxypropyl)trimethylammonium cation	4.4918082	1.3000747	0.048519417
M130T329	.beta.-Homoproline	2.6216583	0.6304035	0.011411409
M809T40_1	1,2-Dioleoyl-sn-glycero-3-phosphatidylcholine	10.302568	1.2673528	0.011526453
M544T183_2	1-Oleoyl-sn-glycero-3-phosphocholine	9.2769265	1.3469773	0.003825061
M719T150	1-Palmitoyl-2-oleoyl-sn-glycero-3-phosphoethanolamine	1.5541315	1.288328	0.021126463
M538T189	1-Palmitoyllysophosphatidylcholine	1.9306271	0.7145894	0.062502812
M482T196	1-Stearoyl-2-hydroxy-sn-glycero-3-phosphoethanolamine	7.8050208	0.6991557	9.36677E-05
M325T390	3.alpha.-Mannobiose	5.1968053	0.5664469	0.066214987
M136T248	Adenine	2.5927429	0.2503528	0.060254528
M205T419	Ala-Asp	3.083012	1.1520022	0.025283968
M83T359_2	DL-2,4-diaminobutyric acid	1.5369914	1.2581143	0.001086958
M182T299_2	L-tyrosine	2.8819903	0.9040982	0.073387516
M337T192	MG (18:2(9Z,12Z)/0:0/0:0)[rac]	1.7152056	0.6612212	0.002381092
M213T429	Pro-Asp	2.337029	0.1343795	0.02772683
M170T110	Pyridoxine	3.0990518	0.6472242	0.000559739
M198T448	sn-Glycerol 3-phosphoethanolamine	1.7596821	1.5846814	0.001913855
M759T119	Thioetheramide-PC	21.472648	0.6559066	0.038089307
M165T299_2	trans-2-Hydroxycinnamic acid	2.655104	0.8982296	0.066511547
	Negative mode			
M136T103	Anthranilic acid (Vitamin L1)	1.7150793	0.6310405	0.042811618
M161T450	D-fructose	1.2309281	0.373283	0.0240985
M217T407	5-L-glutamyl-L-alanine	1.2637296	1.6682473	0.028708157
M132T401	D-aspartic acid	2.6296447	0.8341284	0.003988221
M146T396_2	L-Glutamate	9.0500396	0.7744994	0.00077862

M145T428	L-glutamine	1.0376406	2.3110724	0.003144328
M154T440	L-histidine	1.6697692	0.5418694	9.92781E-05
M115T405_2	Maleic acid	2.432395	0.8241391	0.016376489
M111T90_2	Uracil	6.0099062	0.7865019	0.027036672
M279T46	Linoleic acid	24.368227	0.7933628	0.04328489
M225T50	Myristoleic acid	7.6448941	1.5768305	0.033469822
M281T136	Oleic acid	3.0346879	1.4161755	0.040159628
M464T206	Stearoylcarnitine	1.2876774	0.6693058	0.009535131
M89T158	DL-lactate	2.7614138	1.2852286	0.023776162
M133T405_2	L-malic acid	6.1648396	0.8103438	0.036336098
M391T371	1,3-Dimethyluric acid	1.3349064	0.4648	0.08975302
M141T286	2-Oxoadipic acid	2.6241439	1.5799428	0.020989905
M129T110	Dihydroxyfumarate	3.1782517	1.4452712	0.063942998
M237T158	L-gulonic gamma-lactone	1.1401175	1.3656074	0.097522704
M124T469	Ciliatine	2.3019698	0.5690557	0.001866261
M182T470	Phosphorylcholine	1.6061919	1.3359193	0.006188975
M191T94	D-galactarate	2.1978855	1.3915019	0.085114994
M71T81	Dihydroxyacetone	1.532943	1.2378186	0.020198473
M229T402	Dihydroxyacetone phosphate	1.2081206	1.5976959	0.000652912
M341T391	D-maltose	4.5963694	0.5673535	0.023633155
M299T158	D-ribose	2.0661964	1.8422328	0.010078341
M161T391	D-tagatose	2.8921921	0.5891612	0.044520295
M181T349	D-threitol	1.4067302	0.2310774	0.033091065
M151T243_2	Glycerol	3.3442068	1.2879118	0.071082314
M195T352	L-threonate	4.929838	0.8175265	0.097269923
M563T450	Maltotriose	3.013033	0.315674	0.022151301
M218T275_2	Pantothenate	3.9657181	0.8176015	0.028144499
M71T128	Pyruvaldehyde	2.183915	1.3996283	0.019481389
M225T417	L-carnosine	2.7872299	0.6968553	0.053496342
M182T42	4-pyridoxic acid	2.528534	0.7785241	0.060858688
M166T103	Pyridoxal (vitamin B6)	1.3489646	0.6676288	0.020877455
M346T428	Adenosine 3'-monophosphate	1.0350698	1.2456895	0.017544639
M514T177	Taurocholate	1.9599003	0.6054194	0.044191049
M447T124	1,3,5(10)-Estratrien-3,17.beta.-diol 17-glucosiduronate	1.1307995	3.2797872	0.026675681
M341T450	alpha,alpha-Trehalose	2.0092062	0.3526771	0.02721201
M401T83	Cortisone acetate	1.2093479	1.8860204	0.068255808
M147T157	D-arabinono-1,4-lactone	1.461572	1.2610279	0.090159603
M149T185	D-lyxose	1.4763963	1.2796906	0.084625078
M277T367	gamma-Glutamyl-L-methionine	1.0764832	1.4608338	0.088030166
M175T107	L-ascorbic acid	2.2268894	1.4941233	0.052073182
M213T157	m-Chlorohippuric acid	2.4497175	1.328134	0.016264044
M196T448	sn-Glycerol 3-phosphoethanolamine	1.1823796	1.5308814	0.002699156

GL16 = non-castrated and slaughtered at 16 months of age; YL16 = castrated at birth and slaughtered at 16 months of age; YL26 = castrated at birth and slaughtered at 26 months of age.
